# 

**Published:** 1888-02-04

**Authors:** 


PRICE ONE PENNY.

No. 71, Vol. III—February 4th, 1888.

[Registered at the General Post Office as a Newspaper.]

Per Annum, 6s. 6d., post free.
Monthly Parts, 6d.; post free, 7½d.
Ditto per Ann., 7s. 6d. post free.

